# Projected Shifts in *Coffea arabica* Suitability among Major Global Producing Regions Due to Climate Change

**DOI:** 10.1371/journal.pone.0124155

**Published:** 2015-04-14

**Authors:** Oriana Ovalle-Rivera, Peter Läderach, Christian Bunn, Michael Obersteiner, Götz Schroth

**Affiliations:** 1 International Center for Tropical Agriculture, DAPA program, Cali, Colombia; 2 Humboldt-University of Berlin, Faculty of Agriculture and Horticulture, Berlin, Germany; 3 International Institute for Applied Systems Analysis, ESM program, Laxenburg, Austria; 4 C.P. 513, 68109–971 Santarém, Pará, Brazil; Federal University of Goiás, BRAZIL

## Abstract

Regional studies have shown that climate change will affect climatic suitability for Arabica coffee (*Coffea arabica*) within current regions of production. Increases in temperature and changes in precipitation patterns will decrease yield, reduce quality and increase pest and disease pressure. This is the first global study on the impact of climate change on suitability to grow Arabica coffee. We modeled the global distribution of Arabica coffee under changes in climatic suitability by 2050s as projected by 21 global circulation models. The results suggest decreased areas suitable for Arabica coffee in Mesoamerica at lower altitudes. In South America close to the equator higher elevations could benefit, but higher latitudes lose suitability. Coffee regions in Ethiopia and Kenya are projected to become more suitable but those in India and Vietnam to become less suitable. Globally, we predict decreases in climatic suitability at lower altitudes and high latitudes, which may shift production among the major regions that produce Arabica coffee.

## Introduction

There is a large body of evidence that substantiates global warming, with increases in mean atmospheric and oceanic temperatures [1]. The agricultural sector will face serious challenges in the coming decades due to the sensitivity of crops to water shortages and heat stress [2]. Rising temperatures have already reduced crop quality and increased the pressure of pests and diseases, reducing agricultural production worldwide [3].

Coffee ranks just after oil in its value among traded commodities and is grown in more than 60 tropical countries [4]. Brazil, Vietnam, Indonesia and Colombia together produce more than 65% of the global total [5]. An estimated 25 million farmers produce coffee on over 1 million ha, most of whom are smallholders who depend on coffee for their livelihoods [6]. This web of small coffee farms is important in the economies of some developing countries, for example, coffee contributes 59% of Burundi's export earnings and 17% of Nicaragua’s [5].

Arabica (*Coffea arabica*) and Robusta (*Coffea canephora*) coffees dominate production (5.1 Mt and 3.1 Mt respectively in 2011 [7]). Arabica coffee is mostly grown in tropical highlands and is used in gourmet coffees due to its higher quality, while Robusta coffee is lower quality grown at lower altitudes. Both species originate from Africa.

Climate change brings increased temperature, which reduce growth, flowering and fruiting [8]. It also increases pressure of coffee pests [9,10]. Coffee farmers in Nicaragua report change patterns of seasonal rainfall over the past 20 years, leading to erratic flowering, incomplete maturation and fruit drop [11].

Regional studies of the impact of climate change on Arabica coffee have shown that the suitable area will decrease and move to higher elevations. The studies were for Nicaragua [12], México [13,14], El Salvador, Costa Rica, Honduras and Guatemala [15,16], Brazil, Tanzania and Vietnam [15] and Indonesia [17]. Davis [18] concluded that climate change has reduced and will continue to reduce the production of Arabica coffee in East Africa.

So far no one has studied the effect of climate change on the global distribution of climates suitable to produce Arabica coffee or on the changes within the regions of current production. We modeled the global distribution of climates suited for growing Arabica coffee based on data of where coffee is grown at present. We projected future changes in climatic suitability on the basis of 21 global circulation models (GCMs) for the year 2050s. We highlight relative decreases and increases of climatic suitability for the world's main Arabica-producing regions and countries. We discuss relative shifts in areas suitable to grow Arabica coffee within and among regions that may result from global climate change.

## Materials and Methods

### Coffee distribution data and sample selection

**Fig 1:**
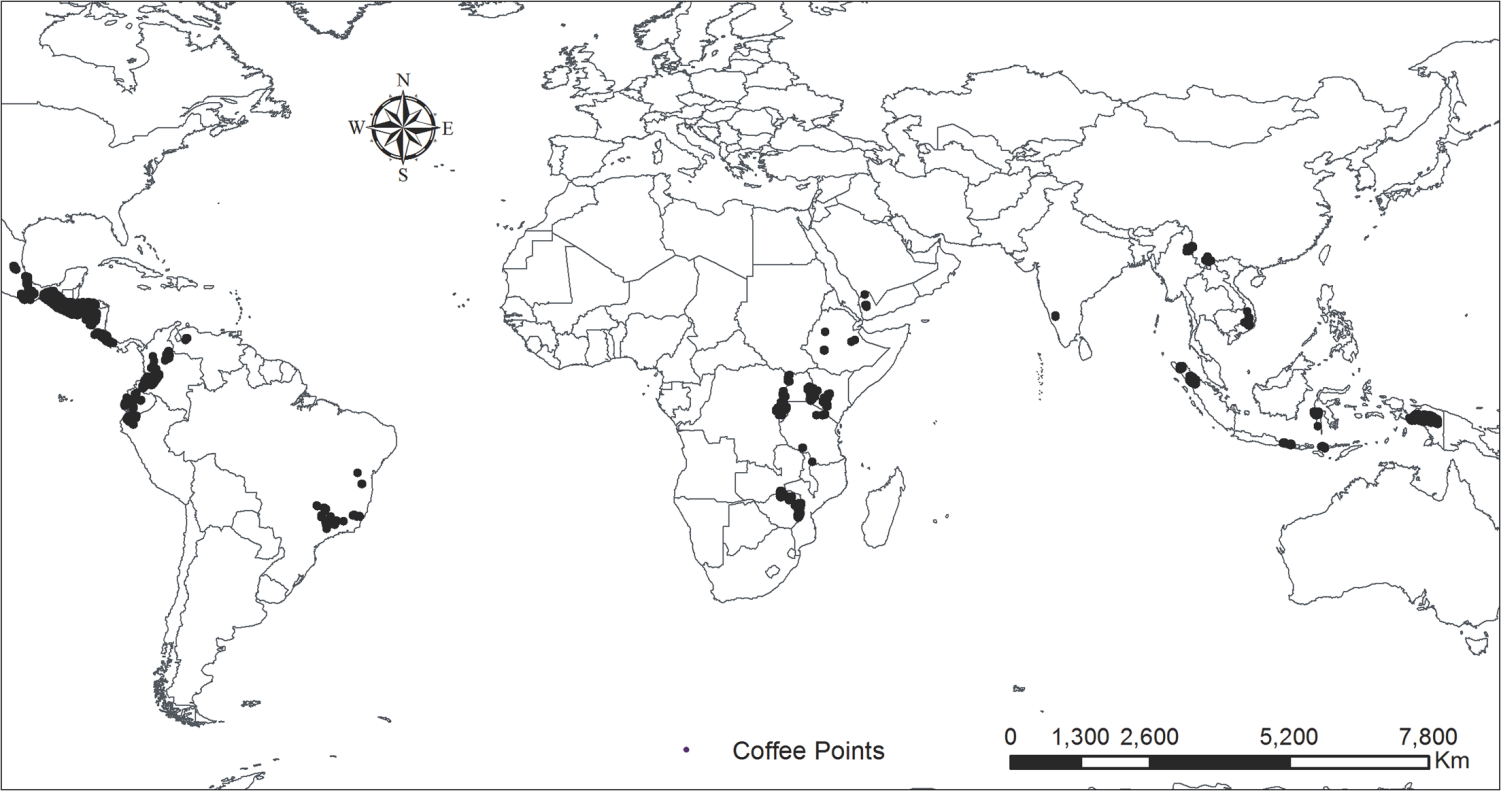
Arabica coffee locations used for the analysis.

We compiled a spatial database of more than 62,000 points that we knew grow Arabica coffee (Fig 1). Most of the data came from projects developed by the International Center for Tropical Agriculture (CIAT) in collaboration with other research centers and producer cooperatives. The sites were in 19 countries: Brazil, Colombia, Ecuador, Mexico, Costa Rica, El Salvador, Nicaragua, Guatemala, Honduras, Ethiopia, Kenya, Rwanda, Burundi, Tanzania, Uganda, Zimbabwe, India, Yemen, Indonesia, and Vietnam. We included additional locations suitable to produce Arabica coffee based on information provided by national coffee research institutes and a literature review.

We then stratified the database based on elevation. The literature review provided a range of elevations suitable to produce Arabica coffee in each country, with lower elevations at higher latitudes. We filtered the 62,000 points to exclude any data from elevations outside these ranges. We used environmental data at a resolution of 2.5 arc minutes, so that we further filtered the database to identify locations at the centroid of each grid pixel. This reduced the dataset to 17,625 location points. The reduced dataset had a range of mean annual temperature 14–26°C, and mean annual precipitation 186–4930 mm. We assumed that sites with annual rainfall less than 750 mm, the minimum required by coffee [19], were irrigated (Table 1), which was outside the scope of the study. We excluded these sites from the analysis.

**Table 1:** Climatic conditions at Arabica coffee locations used in the analysis.

Variable	Unit	Mean	Std. Dev.	Min	Max
**Annual Mean Temperature**	°C	20.74	19	14.20	25.60
**Mean Temperature of Warmest Quarter**	°C	21.98	20	14.70	27.20
**Mean Temperature of Coldest Quarter**	°C	19.07	21	12.10	24.90
**Annual Precipitation**	mm	1875	703	754	4199
**Precipitation of Wettest Month**	mm	315	106	99	755
**Precipitation of Driest Month**	mm	40	49	0	291
**Precipitation Seasonality^1^**	-	67	23	8	114
**Precipitation of Wettest Quarter**	mm	832	277	263	1967
**Precipitation of Driest Quarter**	mm	142	158	2	939
**Precipitation of Warmest Quarter**	mm	516	194	36	1307
**Precipitation of Coldest Quarter**	mm	248	256	2	1332
**Mean Diurnal Range**	°C	11.49	17	77	181
**Isothermality^2^**	-	7.39	8	48	92
**Temperature Seasonality^3^**	-	115.83	657	118	426.2
**Max Temperature of Warmest Month**	°C	28.44	24	19.8	34.9
**Min Temperature of Coldest Month**	°C	12.76	26	4.8	19.9
**Temperature Annual Range**	°C	15.68	30	8.9	25.3
**Mean Temperature of Wettest Quarter**	°C	21.35	20	14.3	26.5
**Mean Temperature of Driest Quarter**	°C	19.83	23	12.1	25.7

^1^Coefficient of Variation

^2^(Mean Diurnal Range /Temperature Annual Range)* 100

^3^standard deviation* 100

We divided the remaining locations according to climate by a principal components analysis. We used the Calinski test (Caliński and Harabasz, 1974, Eq 1) to identify the optimal number of points per cluster and to select 2194 of them, which we call ‘presence locations’.

$$n=\frac{Z^{2}\;pq\;N}{N\;E^{2}+Z^{2}\;pq}$$ 1

Where *n* = Sample size,

*N* = Population size,

*Z* = Z-value (1.96),

*p* = Expected proportion (0.5),

*q* = Complement (1- *p*) and

*E* = Expected Error (±0.05).

Distribution of the presence locations was not uniform across countries or latitudes, which could distort the analysis. We therefore sorted the sample by country. If a country had fewer than 12 data points for each climate category, we selected all of them.

### Climate data

#### Current climate

We used the 19 bioclimatic variables from the Worldclim 2.5 arc minute resolution database [20]. The bioclimatic variables include annual mean temperature and precipitation, and extreme or limiting factors that are ecologically important (Table 1).

#### Future climate

We based estimates of the climate in 2050s on the 21 GCMs used in the Intergovernmental Panel on Climate Change (IPCC) Fourth Assessment Report (AR4) for scenario A2a of the IPCC’s SRES [21].

We downscaled the low-resolution (1–2 arc degrees) of the output of each GCM using the delta method [20,22] to produce an interpolated surface of change in climates. We then applied these interpolated surfaces to WorldClim's baseline 2.5 arc minute surface climate, accounting for differences in the baselines to avoid bias. The method assumes that the relationship between climate variables will be the same the future as now and that changes in climate will only be at coarse scales [22].

### Current and future coffee suitability prediction

We used the MaxEnt algorithm [23], which is easy to use and gives reliable extrapolation [24]. Moreover, the user can derive a suitability function based on point input and background data to characterize the environment in the study region [25]. MaxEnt is especially suited for coffee, which is a climate-sensitive crop [26]. MaxEnt predicted occurrence probabilities that were statistically better than the alternatives CaNaSTA, Domain, and Bioclim. MaxEnt is a proven useful model to analyze the impact of climate change on coffee [14,18,27,28].

MaxEnt output is spatial distribution of probabilities that the climatic environment in a given pixel is suitable for the species in question, here Arabica coffee. We used a suitability threshold of 0.4, which was where MaxEnt showed equal specificity and sensitivity during training, and which indicated habitats that were favorable for the crop [29].

In addition to the 2194 presence locations, we selected a global, random sample of 20,000 pseudo locations where Arabica coffee does not grow. MaxEnt requires these pseudo locations to define the presence region during the training phase.

We trained the MaxEnt algorithm using the whole set of presence locations to obtain the suitability function for Arabica coffee. We then applied the derived suitability function to each of the 21 downscaled GCMs to estimate the global distribution of suitability in 2050s. We used these projections to calculate the median suitability and its quartiles, which represent low, medium, and high scenarios for the impact of climate change on Arabica coffee in 2050s.

### Model validation

To validate the model, we used 25 replicate runs. For each run we selected at random 80% of presence locations (1756) to train the model and the remaining 20% (438) to assess its predictive performance. To assess model skill we used the area under the curve (AUC) of the receiver operating characteristic [30] and the maximum Cohen’s kappa (kmax) [31]. Both are measures of an algorithm’s power to discriminate binary data; using both compensates for weaknesses in one or the other [31].

## Results

The results are based on an analysis of MaxEnt modeling and the output of 21 GCMs as outlined above. They are therefore predictions with a degree of uncertainty rather than forecasts with a statistical error term.

### Parameterization, model validation, uncertainty

**Fig 2:**
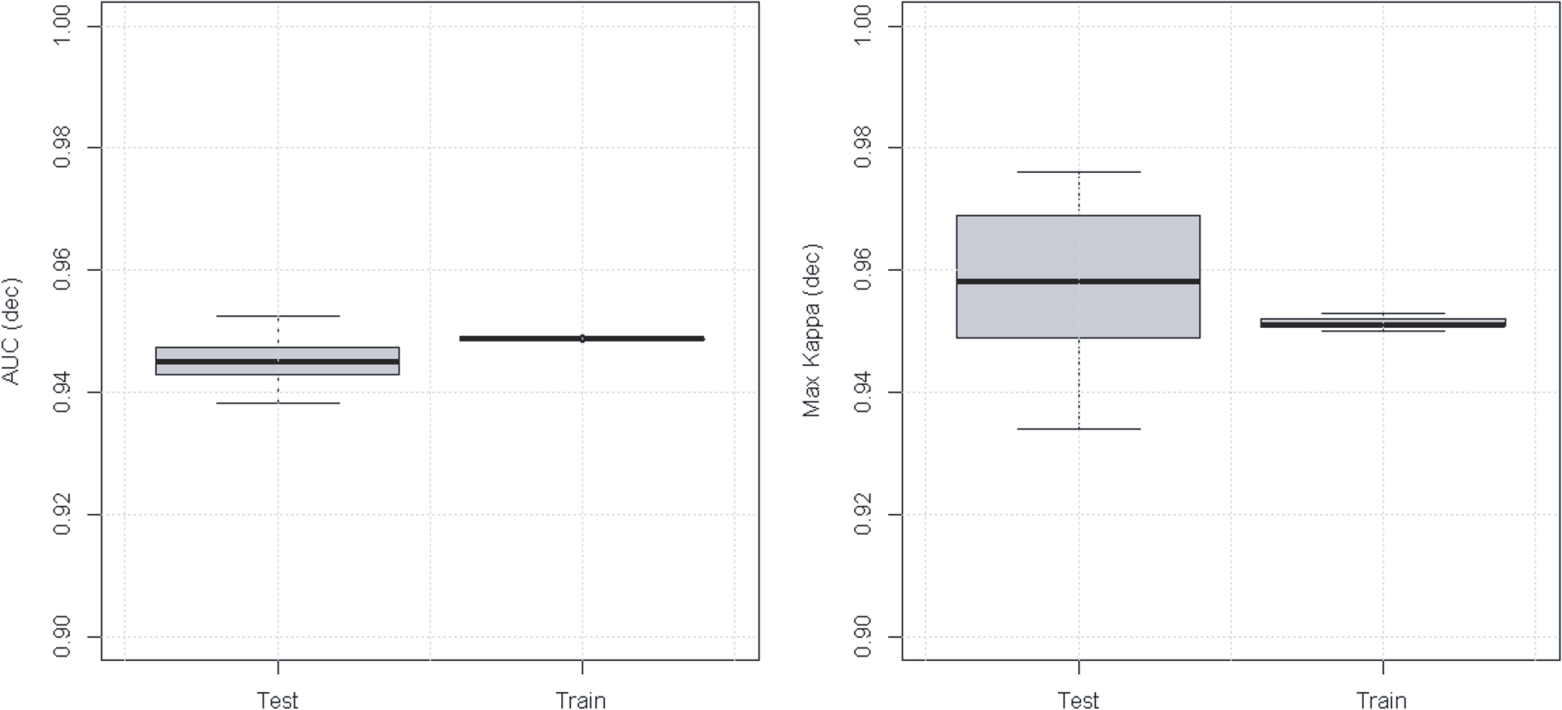
Performance of the MaxEnt model across 25 replicates: (A) AUC, and (B) maximum Cohen’s kappa. The thick black horizontal line shows the median, the box shows the quartiles and the whiskers show 5–95% of the distributions.

MaxEnt performed well with AUC (kappa) values 0.94–0.97 (0.95–0.97) for the test data (20%) and almost no variation for the training data (80%) (Fig 2). MaxEnt’s good performance gave low uncertainties of the baseline. We calculated the median and quartile values of each downscaled cell of the 21 GCMs. We then estimated the uncertainty in their predictions of change in climate suitability for coffee. We did this separately for negative change, no change, or positive change.

### Uncertainties due to multiple GCMs

Comparison of the medians and quartiles gives a measure of the uncertainty between the 21 GCMs’ predictions. The pessimistic scenario predicts that a third of the current coffee area would lose more than 40% climate suitability (Table 2). Nearly half of the current coffee-growing area would lose 20–40% climate suitability, mainly in areas of low–medium elevation. For the median of the GCMs, a quarter of the current coffee-growing area would experience no change, 27% would lose 10–20% suitability, and 37% would lose 20–40%. Under the optimistic scenario, 52% of the current coffee-growing area would experience no change in suitability, and 34% of the area would lose 10–40% suitability, while 6% would become more suitable.

**Table 2:** Changes in coffee crop suitability and scale of uncertainties in climatic models by 2050s.

Projection	Suitability change	Area affected (%)				
		Lose			No change	Gain
		< -40	-20–-40	-10–-20	-10–10	>10
2050	First quartile	33	51	11	4	-
	Median	12	37	27	24	1
	Third quartile	6	20	14	52	6

### Regional climate change and impacts on coffee suitability

**Fig 3:**
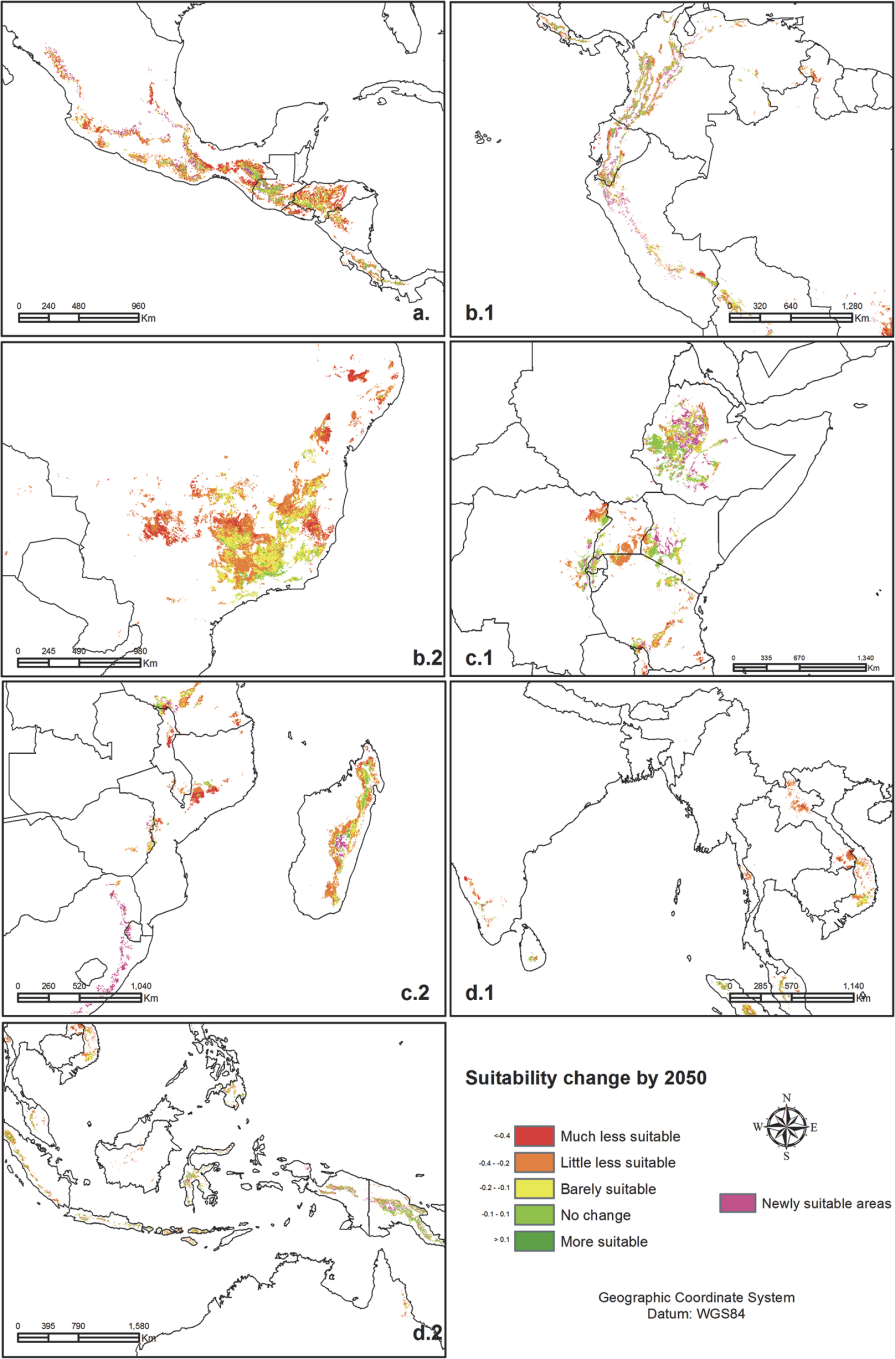
Changes in coffee suitability in 4 coffee growing zones by 2050s. a. Mesoamerica, b.1 - b.2 South America, c.1 – c.2 Africa, d.1 – d.2 Pacific.

We divided the global areas growing Arabica coffee into Mesoamerica, South America, Africa and Asia-Pacific (Tables 3 and 4 and Fig 3). We present the patterns of change by 2050s in climate and suitability of Arabica coffee for these regions.

**Table 3:** Geographic locations and change in climatic characteristics by 2050s.

Region	Latitude	Climate change by 2050s		
		Precipitation (mm)	Dry months (month)	Temperature (°C)
Mesoamerica	10–20° northern	-70	-1	+2
South America	12º northern and 14º southern	+100 - +120	+1 - +2	+2.1
	5º - 27º southern	+273	0	+2.5
Africa	10º northern and 11º southern	+40	-1	+2.1
	12º and 26º south	-20*	-1	+2.1
Asia Pacific	24º and 3º northern	14	0	1.8
	3º northern and 10º southern	120	-	1.7

**Table 4:** Changes in suitability and area (km^2^) of coffee suitability in 4 coffee growing zones by 2050s.

Region	Country	Current Potential area for Coffee (km^2^)	Current potential area for coffee excluding protected area (km^2^)	*Change in suitability by 2050s (excluding protected areas)		
				Average	Minimum	Maximum
**Mesoamerica**	Costa Rica	3,130	2,165	-0.20	-0.55	0.18
	Guatemala	7,385	6,635	-0.19	-0.82	0.18
	Honduras	13,795	12,315	-0.27	-0.67	0.12
	Mexico	30,605	27,430	-0.29	-0.85	0.11
**South America**	Bolivia	9,435	4,915	-0.20	-0.57	0.06
	Brazil	129,335	118,770	-0.25	-0.70	0.13
	Colombia	21,880	18,970	-0.16	-0.61	0.21
	Ecuador	8,245	7,345	-0.20	-0.72	0.14
	Peru	10,480	7,390	-0.20	-0.73	0.16
**Africa**	Burundi	525	495	-0.09	-0.32	0.09
	Ethiopia	40,800	35,095	-0.11	-0.61	0.23
	Kenya	10,380	9,550	-0.12	-0.40	0.17
	Rwanda	1,610	1,570	-0.09	-0.30	0.10
	Uganda	8,070	7,550	-0.25	-0.46	0.06
	Tanzania	18,315	15,710	-0.22	-0.84	0.11
	Zambia	20	10	-0.09	-0.15	-0.03
**Pacific**	India	2,705	2,110	-0.28	-0.69	0.04
	Vietnam	6,165	4,730	-0.25	-0.58	0.14
	Indonesia	36,510	22,740	-0.18	-0.62	0.16
	Papua New Guinea	14,690	14,310	-0.09	-0.54	0.13
***TOTAL***		***374*,*080***	***319*,*805***	***-0*.*19***	***-0*.*59***	***0*.*12***

*Threshold (0.4).

#### Mesoamerica

Located latitude 10–20°N on the narrow isthmus of Central America, this zone is characterized by rugged volcanic mountains up to 4000 masl with different levels of suitability for Arabica coffee. The GCMs predict that annual precipitation would only decrease from 1670 mm now to 1600 mm, and the number of dry months (defined as months with less than 100mm of rainfall) would decrease from 6 to 5 months. Maximum and mean temperatures would increase by 2°C.

Higher temperatures would move the climates suitable for Arabica coffee from the current 400–2000 masl to 800–2500 masl. Nicaragua and El Salvador, which do not have high mountains, would be most affected. Guatemala, Mexico, Honduras, and Costa Rica would gain suitability at elevations 1500–2500 masl, which could compensate in part for losses at lower altitudes. Land at higher altitudes is often forested, so that we expect increased land-use pressure on high-altitude forests [14]. Mesoamerica would confront an average decrease in the area suitable for Arabica coffee up to 30%, with largest losses for Mexico (29%) and smallest losses for Guatemala (19%) (Table 4).

#### South America

Coffee in South America grows in the Andes of Peru, Ecuador and Colombia, 12°N–14°S latitude, and in the eastern highlands of Brazil, 5–27°S latitude. In the Andes, whose peaks rise above 5000 masl, coffee grows above 500 masl. In Brazil, part of the zone is subtropical, producing quality coffee at low elevations.

Precipitation in the Andes would increase 100–170 mm, with more rain during the wet season. In contrast, precipitation in Brazil would decrease by 50 mm in the dry season. The number of dry months in the Andes would increase from 1 month to 2 months, while in Brazil the dry season would be unchanged at 6 months. Maximum, mean, and minimum temperatures would increase in both the Andes and in Brazil.

The range of elevations suitable for Arabica coffee in the Andes is predicted to move from currently 500–1500 masl to 1000–2800 masl. Areas below 1800 masl in all three countries would become less suitable. Peru, Colombia and Ecuador, however, will gain some suitable areas at higher elevations. In Brazil, there would be a shift in suitable climates from the current 400–1500 masl to 800–1600 masl. Brazil has no high elevations and grows large areas of Arabica coffee at low elevations, which are predicted to suffer substantial losses in suitability for coffee. Only a few farms at higher elevations could expect improved suitability. Overall, the Andes countries would lose 16–20% of the current area suitable for Arabica coffee while Brazil would lose 25%. We emphasize that these figures are for where Arabica coffee grows now. Although losses in the Andes will be less than in Brazil, the difference is that there will be alternatives in the Andes that Brazil will not have.

#### Africa

There are two sub-regions of Africa that grow Arabica coffee. The East African sub-region is located 10°N–11°S latitude at high elevation in the Great Rift Valley, which is the centre of origin of *Coffea arabica*. The southern Africa and Madagascar sub-region is in the mountains at latitudes 10°–26°S. There is no Arabica coffee grown above 2500 masl in the whole region.

In the East African sub-region annual rainfall is predicted to increase somewhat, from 1400 mm to 1440 mm, and the dry season to decrease from 5 to 4 months. In Southern Africa and Madagascar, rainfall is predicted to become only a little more seasonal with a slight increase in the wettest month and a slight decrease during the driest month. The dry season would decrease from 7 to 6 months. Maximum, mean, and minimum temperatures are predicted to increase by about 2°C throughout the region.

In East Africa, climates suitable for Arabica coffee are predicted to shift from 400–2000 masl to 800–2500 masl. There would be little change in suitability of the areas in Ethiopia, Kenya, Rwanda, and Burundi that currently grow Arabica. There may be gains as areas at higher elevations (1500–2400 masl) become more suitable. Tanzania and Uganda would lose suitable area at elevations below 1400 masl. In Southern Africa and Madagascar, the suitable climates would shift upward from 500–1700 masl to 700–2000 masl, resulting in losses of suitable area at lower elevations, especially in Zimbabwe, as its growing area are at low altitudes.

#### Asia-Pacific

In the Asia-Pacific region, the areas that grow Arabica coffee are in India, Indochina, Indonesia and the Pacific Islands. The areas in India and Indochina are at latitude 3°–24°N, in southern India at elevations up to 2500 masl and in Vietnam at elevations less than 2000 masl. The coffee areas in Indonesia and the Pacific islands are at latitudes 3°N–10°S.

In India and Indochina, precipitation is predicted to increase only slightly with little change in dry season rainfall or duration. Only small changes in rainfall are predicted in Indonesia and the Pacific Islands. Annual rainfall would increase somewhat (from 2670 mm to 2750 mm) and become slightly more seasonal with a little more rain during the wettest month (from 331 mm to 358 mm) and a little less rain during the driest month (from 123 mm to 115 mm). Maximum, mean and minimum temperatures are predicted to increase throughout the region.

Suitable climates for Arabica coffee in India and Indochina would shift upward from the current 400–1500 masl to 700–1800 masl. India and Laos would experience a loss of suitability below 1200 masl. In Indonesia and the Pacific islands, suitable climates would also shift upward from the current 500–2000 masl to 800–2300 masl. Indonesia would likely suffer a reduction of 21–37% in the area suitable to produce Arabica coffee, while Papua New Guinea with its high elevations would be less affected. Using a similar modeling approach [17] have shown that the area suitable for growing Arabica coffee in Indonesia would be smaller in 2050s than it is now, but that the suitable area in 2050s would still be larger than the area currently used for growing the crop, suggesting that through a shift in production areas current total production levels might be maintained.

### Global overview of suitability change by 2050s

**Fig 4:**
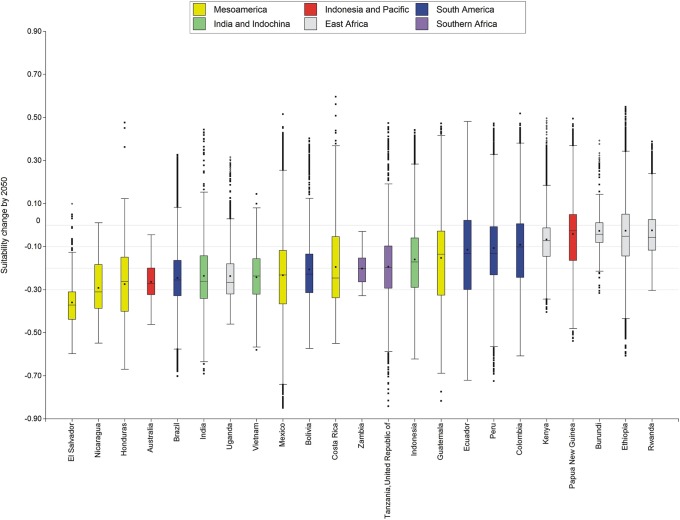
Suitability change in main *Coffea arabica* growing countries by 2050s. The dot represents the mean, the line the median, and the limits of the boxes are the 0.25 and 0.75 quartiles, while the extremes are the 0.05 and the 0.95 quartiles and the dots beyond the outliers.

A global comparison of countries (Figs 3 and 4) shows that climates with highest suitability for Arabica coffee are currently located between 600–1900 masl. Higher and lower elevations are less suitable because they are too cold or too hot, respectively. The actual limits depend on the latitude and topography of the specific country.

By 2050s, it is predicted that global temperatures would increase by 2°C together with some increased seasonality of precipitation. These changes would reduce climatic suitability for Arabica coffee at low elevations and increase suitability of higher areas. The net effect is that coffee farming will tend to move uphill.

The predicted changes in coffee suitability are directly linked to latitude. Higher temperatures would cause areas growing Arabica coffee within 5°–10° of the equator at elevations less than 1000 masl to lose climatic suitability. Changes in annual precipitation and its seasonality would have little effect.

## Discussion

### Global and regional climate change impacts

Our analysis shows that the impacts of climate change on climate suitability of Arabica coffee would be very variable at both the national and global levels. Overall, the impact of climate change in all producing countries is predicted to be negative, although within each country it would vary a lot. Some areas would lose suitability while others would gain from increases in temperature and perhaps in rainfall.

All the coffee-producing countries in America, Africa, Asia, and Oceania would maintain some suitability for growing Arabica coffee. Colombia, Ethiopia, Indonesia, Mexico, and Guatemala have extensive areas of land at high elevation that receive sufficient rainfall. An upward move of their coffee-growing areas could moderate the overall impact of climate change on their countries’ coffee industry. An important proviso is that the areas at higher elevation are available for conversion to coffee farms, are accessible, have suitable soil conditions, and whose current or future inhabitants are willing to grow Arabica coffee rather than other crops [14,17]. Very often, these conditions may not all come together, with the consequence that Arabica coffee production may locally decline.

The regions where Arabica coffee would be least affected by higher temperatures are East Africa with the exception of Uganda and Papua New Guinea in the Pacific. Mesoamerica would be the most affected region, specifically Nicaragua and El Salvador. Since Arabica coffee is an important export of Mesoamerica, we expect severe economic impacts here. As previously suggested by Zullo [32], strongly negative effects of climate change are also expected in Brazil the world's largest Arabica producer, as well as India and Indochina. Regions predicted to suffer intermediate impacts include the Andes, parts of southern Africa and Madagascar, and Indonesia, with significant differences among islands [17].

The range of elevation of current coffee-growing areas in each country, together with their topography, will determine the overall impact of climate change on current farms. They will also determine whether farm expansion at higher elevations can compensate for lost suitability at lower elevations. The scenario of Arabica coffee migrating to higher elevations, as temperatures increase is unlikely to be modified by changes in precipitation, which were mostly minor. The largest impacts are to be expected from precipitation changes during the dry season, but there is much uncertainty in their prediction.

The geographically differentiated impacts of climate change may alter the relative importance of coffee from different countries in the global market. Regionally, Guatemala and Costa Rica may compensate for neighboring Nicaragua and El Salvador as their supply volumes and quality decline. On a continental scale, the Andean producers could benefit from a decline in coffee volume and quality in the more severely affected Mesoamerica and Brazil. Globally, there could be a shift in production from more-affected Latin America to less-affected East Africa and Indonesia. Although no country would experience improved climatic suitability, there may be countries less negatively affected by climate change than their competitors on the global Arabica coffee market [17,33].

### Implications for adaptation strategies

Earlier studies highlighted the strong local dimension needed to adapt to climate change. Climate change will have different impacts and producers will have different vulnerabilities at small scales in the mountains where Arabica coffee grows [11,14,17,34]. Our results confirm this, predicting extreme variability in the impacts of climate change within each country (see Fig 3).

Here we have highlighted the global dimension and scale of climate change with its pronounced variation of impacts on Arabica producer countries at regional, continental, and global scales. These show that adaptation strategies are required at all levels.

Some countries, such as in Mesoamerica, will lose competitiveness on global markets for quality coffee. They may need to diversify into other products to prevent adverse effects on their rural economies [28]. Other regions such as the Andes, East Africa and Indonesia may take advantage of new market opportunities. But they may require specific policies and strategies to ensure that expansion of coffee farmlands takes place in climatically, pedologically and ecologically suitable areas [17].

Overall there will be a need for high-quality varieties of Arabica coffee that are better adapted to higher temperatures. This must be a priority for plant breeders in the coming decades.

Our global analysis provides a broad classification of countries as either severely or less severely affected by climate change. In terms of production of Arabica coffee, however, each region, and each country within it, will itself be a mosaic of situations. Some areas will become more suitable while others will lose suitability. This calls for approaches at the local scale to help farmers to adapt to climate change [11].
